# Green cabbage supplementation influences the gene expression and fatty acid levels of adipose tissue in Chinese Wanxi White geese

**DOI:** 10.5713/ab.22.0345

**Published:** 2023-05-02

**Authors:** Bin Wang, Zhengquan Liu, Xingyong Chen, Cheng Zhang, Zhaoyu Geng

**Affiliations:** 1 Department of primary education, Tongcheng Teachers College, Tongcheng 231400, China; 2 Anhui Province Key Laboratory of Local Livestock and Poultry, College of Animal Science and Technology, Anhui Agricultural University, Hefei 230036, China; 3 College of Science, University of the Philippines Baguio, Baguio City, Philippines

**Keywords:** Fattening, Fatty acid, Green Cabbage, Stearoyl-CoA Desaturase, Wanxi White Goose

## Abstract

**Objective:**

Dietary green cabbage was evaluated for its impact on fatty acid synthetic ability in different adipose tissues during fattening of Wanxi White geese.

**Methods:**

A total of 256 Wanxi White geese at their 70 days were randomly allocated into 4 groups with 4 replicates and fed 0%, 15%, 30%, and 45% fresh green cabbage (relative to dry matter), respectively, in each group. Adipose tissues (subcutaneous and abdominal fat), liver and blood were collected from 4 birds in each replicate at their 70, 80, 90, and 100 days for fatty acid composition, relative gene expression and serum lipid analysis. Two-way or three-way analysis of variance was used for analysis.

**Results:**

The contents of palmitic acid (C16:0), palmitoleic acid (C16:1), linoleic acid (C18:2), and alpha-linolenic acid (C18:3) were feeding time dependently increased. The C16:0 and stearic acid (C18:0) were higher in abdominal fat, while C16:1, oleic acid (C18:1), and C18:2 were higher in subcutaneous fat. Geese fed 45% green cabbage exhibited highest level of C18:3. Geese fed green cabbage for 30 d exhibited higher level of C16:0 and C18:0 in abdominal fat, while geese fed 30% to 45% green cabbage exhibited higher C18:3 in subcutaneous fat. The expression of *Acsl1* (p = 0.003) and *Scd1* (p<0.0001) were decreased with green cabbage addition. Interaction between feeding time and adipose tissue affected elongation of long-chain fatty acids family member 6 (*Elovl6*), acyl-CoA synthetase long-chain family member 1 (*Acsl1*), and stearoly-coA desaturase 1 (*Scd1*) gene expression levels (p = 0.013, p = 0.003, p = 0.005). Feeding time only affected serum lipid levels of free fatty acid and chylomicron. Higher contents of C16:0, C18:1, and C18:3 were associated with greater mRNA expression of Scd1 (p<0.0001), while higher level of C18:2 was associated with less mRNA expression of Scd1 (p<0.0001).

**Conclusion:**

Considering content of C18:2 and C18:3, 30% addition of green cabbage could be considered for fattening for 30 days in Wanxi White geese.

## INTRODUCTION

Fatty acid composition in adipose tissues (including subcutaneous fat, abdominal fat, etc.) depends primarily on the age, gender, and breed of animals. However, diet is one of the most important extrinsic factors affecting fatty acid composition [1], while its influence also depends on the duration of feeding time [2]. Animals, such as cattle, beef [3], goat, etc., usually produce fat with more yellow color under extensive grass-based feed condition [4,5].

It is stated that the fatty acid content would be enhanced by fresh green forage with a predominance of oxidative fiber that has a favorable effect on the deposition of polyunsaturated fatty acid (PUFA) [5,6]. The fatty acid profile of animal products has been a primary area of consumer concern. Adequate intake of a proper polyunsaturated fatty acid/saturated fatty acid ratio (PUFA/SFA), may reduce the risk of lifestyle diseases such as coronary artery disease, hypertension, diabetes, inflammation and immune disorders. Goose is a rich source of high-quality protein with small amount of collagen (0.39% to 0.91%) [7], high level of unsaturated fatty acid and low cholesterol (52 to 76 mg/100 g) content [8]. It is also a rich source of Vitamins A, B1, B2, B3 [9,10], and B6 as well as minerals Ca, Cu, Fe, Mg, P, K, and Na [11]. Geese display a high resistance to disease and a high feed conversion ratio when fed diets with certain content of raw fiber [12]. However, few studies were done on the production and composition of fatty acids in geese when fed green fresh forage.

Fatty acid synthesis mainly relies on a series of catalyzes. For those essential fatty acids such as C18:2 and C18:3, gene expression level would directly affect their synthesis and then the body metabolism. Stearoyl-CoA desaturase (SCD1), removes the hydrogen atoms between C-9 and C-10 position [13] and is mainly expressed in liver and adipose tissues, and could be significantly induced by a high-carbohydrate diet [14]. However, the content of PUFAs, such as linoleic acid and alpha-linolenic acid, inhibit Scd1 mRNA transcription [15]. Long chain fatty acid-CoA ligase (ACSL1) converts free long-chain fatty acids into fatty acyl-CoA esters and thereby plays a key role in lipid biosynthesis and fatty acid degradation [16]. Elongation of long-chain fatty acids family member 6 (ELOVL6) is mainly involved in the elongation of saturated and monounsaturated fatty acids with less than 16 carbons to C18 and does not possess the capacity to elongate beyond C18 [17,18]. More importantly, Elovl6 is ubiquitously expressed in tissues with high lipid content, especially adipose tissues.

For many years, goose products from China accounted for more than 90% of global production. Goose consumption in China varied from soup, roast, braise, etc., depending on a certain abdominal or subcutaneous fat content. Wanxi White geese, a worldwide well-known Chinese indigenous breed, usually undergoes fattening during their 70 to 100 d of age. The purpose of this research is to evaluate the impact of green forage on fatty acid production and composition, and the expression level of fatty acid synthesis related genes mentioned above at different adipose tissues during fattening in Wanxi White geese.

## MATERIALS AND METHODS

### Animal care

All animal procedures were performed in accordance with guidelines developed by the China Council on Animal, Care and protocols were approved by the Animal Care and Use Committee of Anhui Agricultural University (SYDW-P2016 0506006).

### Grouping and treatment of geese

A total of 240 genetically unrelated and sexually mixed Wanxi White geese obtained from Anhui Wanxi White goose preservation, were reared on a floor system in open-sided houses and obtained the same brooding feed before 28 days of age, and then fed growing diet till 70 days of age. All geese were fed a commercial maize-soybean-based diet containing 155 g crude protein/kg feed with a metabolizable energy content from 15.11 to 15.89 MJ/kg feed (Table 1). At 70 d of age, geese were individually weighed, and those within a similar BW range were randomly allocated into four pens with 60 per pen to avoid body weight interference. Each pen was divided into 3 parts as 3 replicates and contained on the same side a 3×3 m outside ground area with a water bath [19].

### Experimental design and diets

The experiment was designed to evaluate 4 levels of fresh green forage (green cabbage) supplementation (0, 15, 30, and 45 percent of green cabbage relative to dry matter). On d 70, birds in group I were fed basal diet, group II fed basal diet and 15% fresh green cabbage, group III fed basal diet and 30% fresh green cabbage, and group IV fed basal diet and 45% fresh green cabbage. The fresh green cabbage was collected from the local farm in each morning and was cut into 10 cm pieces.

### Sample collection

On d 70, 80, 90, and 100 time points, 5 geese from each replicate at each time point were randomly selected for bleeding and then euthanized for liver, subcutaneous and abdominal fat collection after a 12 h fast. Subcutaneous adipose tissue, abdominal fat and liver were first collected and stored in RNALater (TianGen, Beijing, China) for RT-PCR quantification. After the body temperature was completely cooled down, adipose tissue from the same part was collected again and then immediately stored at −20°C for fatty acid composition analysis.

### Blood serum separation and lipid content detection

Measurements of very low density lipoprotein (VLDL), low density lipoprotein (LDL), high density lipoprotein (HDL), triglycerides (TG), total cholesterol (TC), free fatty acid (FFA), and chylomicron (CM) were carried out by the ELISA method using an automatic ELISA analyzer (Thermo Multiskan MK3; Thermo Finnpipette; Thermo Fisher, Waltham, MA, USA). The analyzing kits used were EIA06938Go, EIA05953Go, EIA05782Go, EIA06583Go, EIA06607Go, EIA05651Go, and EIA07354Go, respectively, which were all bought from Wuhan Xinqidi Biological Technology Co., Ltd (Wuhan, China).

### RNA isolation and reverse transcription

Total RNA was extracted from adipose tissue samples by using a commercial kit (TransGen Biotech, Beijing, China) according to the instructions of the manufacturer. Ribonucleic acid concentrations were measured by spectrophotometry using a DU 730 (Beckman, Brea, CA, USA) and their integrity was checked by electrophoresis. After DNase treatment (Takara, Takara Clontech, Beijing, China), 5 μg of total RNA was reverse-transcribed using RNase reverse transcriptase (TransScript RT/RI Enzyme Mix) and random primers (Anchored Oligo(dT) Primer (0.5 μg/μL).

### Determination of mRNA levels by real-time reverse transcription

Complementary DNA (cDNA) was synthesized from 1μg total RNA using the cDNA synthesis kit (Transcript cDNA Synthesis Kit; Transgene, Beijing, China) according to the manufacturer’s recommended protocol. To ensure that there was no possible genomic DNA contamination, negative controls for all samples were also prepared by performing reverse transcription without enzyme mix. Each of the cDNA samples was diluted 1:10 before quantitative real-time polymerase chain reaction (qRT-PCR), and 2 μL diluted cDNA was used per qRT-PCR reaction. All qRT-PCR reactions were performed using the AceQ qPCR SYBR Green Master Mix (Vazyme Biotech Co., Nanjing, China). Each reaction consisted of 2 μL diluted cDNA, 0.4 μL forward primer (10 μM), 0.4 μL reverse primer (10 μM) (Table 2), 10 μL AceQ qPCR SYBR Green Master Mix, 6.8 μL ddH2O. The following PCR conditions were used: 95°C for 10 min for denaturation, followed by 40 cycles of 95°C for 10 s, 53.5°C to 60.0°C (depending on gene, see Table 1) for 1 min, and 72°C for 30 s. After 40 cycles, the reaction volume was held at 72°C for 10 min, and then at 4°C for preservation. All qRT-PCR was carried out using an ABI 7500 thermo cycler (Thermo Fisher, USA). Beta-actin was chosen as a reference. The amplification products for all cDNA were checked by electrophoresis and exhibited the expected size. For each sample (n = 15), the cDNA of genes under study were amplified in triplicate in the same run. Each PCR run included a non-template control and triplicates of control and samples. Gene expression was analyzed using the 2^−ΔΔCT^ method.

### Fatty acid content and composition analysis

The fatty acid profile was determined after the homogenized fat samples were defrosted. A 2 g sample was extracted by using chloroform:methanol (2:1, v/v) solution according to Folch’s method and esterified with methyl alcohol (93%) containing HCL (3%), then applied to diethyene glycol succinate column (DB-WAX, 0.25 mm×0.25 mm; Agilent Technologies Inc., Santa Clara, CA, USA) and were detected with FID by gas chromatography (Agilent GC7890A; Santa Clara, USA). Proportions of fatty acids are reported as percentages of total fatty acids by mass [20].

### Statistical analysis

The fatty acids content and gene expression level were analyzed by three-way analysis of variance (ANOVA) using the general linear model (GLM) procedure of SAS (SAS9.4, Cary, NC, USA) with the main factors of group, feeding time and tissue. The serum lipid contents were compared by two-way ANOVA using the GLM procedure of SAS (SAS9.4, Cary, NC, USA). Fisher’s protected least significant difference (LSD) was utilized to separate means when a significant main effect was observed or to compare subclass means when main effects or their interactions were significant. Partial correlation analysis was conducted using Proc GLM to analyze the correlation between detected fatty acids and related gene expression and serum lipid levels in each tissue. Stepwise regression analysis was done to predict the main factors contributing to fatty acids composition. Statements of significance were based on p<0.05. Before statistical analysis, arcsin transformation was made for alpha-linolenic acid (C18:3) output for its low percentage.

## RESULTS

### Fatty acids composition in subcutaneous and abdominal adipose tissues

Adipose tissues collected on d 100 exhibited higher palmitic acid (C16:0) (p<0.0001), linoleic acid (C18:2) (p<0.0001), and alpha-linolenic acid (C18:3) (p<0.0001), but a lower content of oleic acid (C18:1) (p<0.0001), as compared to other three time points (Table 3). Green cabbage addition did not affect C16:0, palmitoleic acid (C16:1), C18:0, and C18:1, however, geese fed higher content of green cabbage exhibited a higher level of C18:3 (p = 0.003). The fatty acid composition also exhibited differences among adipose tissues. Palmitic acid (C16:0) and stearic acid (C18:0) were higher in abdominal fat, and less in subcutaneous fat (p<0.0001). While subcutaneous fat exhibited higher C16:1, C18:1, and C18:2, as compared to abdominal fat (Table 3).

Abdominal fat exhibited higher C16:0 content after fattening for 20 or 30 d as compared to subcutaneous tissue with any time of fattening (p<0.0001; Table 4). Geese fed green cabbage for 30 d exhibited higher C16:1 content in subcutaneous adipose tissue as compared to geese fed green cabbage at any other times (p = 0.028). Geese fed green cabbage for 30 d exhibited the highest C18:0 content in abdominal fat and the lowest C18:0content in subcutaneous fat as compared among other groups (p<0.0001). The C18:1 level exhibited lowest in abdominal of geese fed green cabbage for 30 d (p = 0.0015). Geese fed green cabbage for 30 d exhibited the highest level of C18:2 in subcutaneous fat, and then the abdominal fat. In addition, geese fed green cabbage for 20 d also exhibited a relatively high level of C18:2 in subcutaneous fat. Geese fed 45% green cabbage for 30 d exhibited the highest level of C18:3 in subcutaneous fat as compared to all the other groups (p = 0.043). The C18:3 detected in abdominal fat from geese fed 45% green cabbage, or from geese fed 30% green cabbage also exhibited a relatively higher level as compared to geese fed 0% to 15% green cabbage (p = 0.043; Table 4).

### Fatty acids synthesis related gene expression

The *Elovl6* and *Acsl1* gene expression level was down regulated with the feeding time (p = 0.013, p<0.0001; Table 5). Green cabbage addition only affected the *Acsl1* gene expression, with the highest expression level in the control group and lower expression in the 30% green cabbage feeding group (p = 0.024). The expression of Acsl1 and Scd1 were higher in subcutaneous as compared to abdominal adipose tissue (p< 0.0001, p = 0.004; Table 5). The interaction between green cabbage and feeding time did not affect gene expression level (data not shown).

The interactions between adipose tissue and feeding time affected *Elovl6*, *Acsl1*, and *Scd1* gene expression (p = 0.013, p = 0.003, p = 0.005; Table 6). Subcutaneous fat of geese from control group or abdominal fat from geese fed green cabbage for 10 d exhibited the highest expression level of Elovl6 as compared to all other groups (p = 0.013). Subcutaneous fat of geese from control group or from geese fed green cabbage for 10 d exhibited a higher expression level of Acsl1 as compared to other groups (p = 0.003). Geese from control group exhibited the highest expression level of Scd1 without considering adipose tissue (Table 6). The interactions between green cabbage and adipose tissue, or between green cabbage and feeding time exhibited no significant differences on the three genes expression level. The cross interactions among feeding time, green cabbage, and adipose tissue exhibited significant differences on the expression of Elovl6 mRNA level (p = 0.051). Birds fed 0%, 15%, or 45% green cabbage for 30 d exhibited the lowest expression level of Elovl6 from abdominal adipose tissue as compared to birds fed 0% or 15% green cabbage for 10 to 20 d from abdominal or subcutaneous tissue. While birds from control group after 10 d of green cabbage feeding exhibited the highest expression level of Elovl6 (p = 0.051; Table 6).

### Blood serum lipid parameters among groups

Serum VLDL, LDL, HDL, TG, and TC all exhibited no differences among feeding time, cabbage percentage, and their interactions in Wanxi White geese. While feeding time affected VLDL, HDL, FFA, and CM content, with the highest in control group, and no difference among all other three feeding groups (p = 0.006, p = 0.014, p = 0.0003, p = 0.001; Table 7).

### Correlation analysis between fatty acids with serum lipid parameters and gene expression levels

The higher C16:0 content was associated with greater serum LDL (p = 0.005), HDL (p = 0.028), TC (p<0.0001) level, and greater Scd1 mRNA expression (p<0.0001), however, with lower serum FFA (p = 0.018) and CM (p = 0.004) level. The higher C16:1 content was only associated with higher Elovl6 mRNA expression (p = 0.047). The content of C18:0 was negatively associated with serum VLDL level (p = 0.041). The content of C18:1 was positively associated with serum HDL (p = 0.013) and TC (p = 0.0001) level, and Scd1 mRNA expression level (p = 0.0001), and negatively associated with serum TG level (p = 0.012). The percentage of C18:2 and C18:3 exhibited high correlations with all the serum parameters detected in this experiment. However, when the C18:2 was positively associated with serum VLDL (p = 0.005), TG (p = 0.001), FFA (p<0.0001), and CM (p<0.0001) level, the C18:3 was negatively associated with these parameters (p< 0.0001), while the C18:2 negatively associated with serum LDL, HDL, TC, and the Scd1 mRNA expression level (p< 0.0001), the C18:3 would positively associate with these parameters (p<0.0001; Table 8).

The total variation in final C16:0 and C18:1 content was explained by the fattening time with 16.91% and 5.68%, respectively.

## DISCUSSION

It is now world-widely accepted that a reduction in SFA and an increase in PUFA would be helpful to human health [21,22]. Consumers growing interest in grass-fed animal products is based on a significant increase in the improvement of fatty acid composition, like the enhancement of total conjugated linoleic acid (C18:2), and omega-3 (n-3) fatty acids on a g/g fat basis [23]. In this experiment, the fatty acids content of C16:0, C16:1, C18:2, and C18:3 all exhibited feeding time-related increased deposition, with the highest level after 30 days fed green cabbage. These results were consistent with previous research suggesting that the linoleic acid isomers and PUFA could be promoted in grass-fed cattle or beef [24–26].

While the ration of green cabbage added in the feed only affected linoleic acid (C18:2) and alpha-linolenic acid (C18:3), together described as the essential PUFA, suggested that 30% to 45% green cabbage relative to dry matter produced the highest level of linoleic acid (C18:2) and alpha-linolenic acid (C18:3). Garcia et al [26] fed beef with pasture and found a higher content of alpha-linolenic acid (C18:3) as compared to beef fed corn-soybean, while no change was detected in linoleic acid (C18:2) content among groups. Fat deposition and composition may vary according to location, which means fat composition usually changes and will not deposit to the same degree [27]. In this research, when the different adipose tissue sites were compared with each other to ascertain any site-specific differences in fatty acid composition, abdominal fat was found to be the most saturated with palmitic acid (C16:0) and stearic acid (C18:0) at the highest level among fat tissues, whereas in subcutaneous, unsaturated fatty acid, palmitoleic acid (C16:1), oleic acid (C18:1), linoleic acid (C18:2) and alpha-linolenic acid (C18:3), were the highest level among fat tissues. The correlation analysis within fatty tissues also exhibits quite differently among tissues. Garaulet et al [28] analyzed the fatty acids composition of adipose tissue in an obese Mediterranean population, and found no significant differences between the different adipose regions with regard to total PUFA content further suggesting the linoleic acid (C18:2) and alpha-linolenic acid (C18:3) could not be synthesized de novo. Therefore, the diet determines the concentrations of fatty acids in adipose tissue. Some tests suggest a homogenous composition of fat throughout the body [29], however, Xiong et al [30] detected a higher content of PUFA in subcutaneous fat, which might explain why the superficial or subcutaneous fat is softer than the deeper fat. The difference in composition between subcutaneous, abdominal fat, and omental fat might indicate that the relations of these different adipose regions with lipolytic activity also differ.

There were only differences among palmitoleic acid (C16:1), linoleic acid (C18:2) and alpha-linolenic acid (C18:3) detected when considering the interaction between feeding time and green cabbage ratio, which suggested that green cabbage and feeding time have a greater impact on unsaturated fatty acids. In addition, geese fed 15% to 45% green cabbage deposited much more unsaturated fatty acid after 30 days green forage feeding. It is worth mentioning that after 30 days of green cabbage addition, the greatest deposits of linoleic acid (C18:2) was in the subcutaneous and in mesentery. These results further suggest that geese fed for a long time on green forage during its fattening period dos much better for unsaturated fatty acid deposition.

The SCD1 specifically catalyzes the Δ9-cis desaturation of saturated fatty acyl-CoAs and is expressed in liver and adipose tissues. The preferred substrates for SCD1 are palmitoyl- and stearoyl-CoA, which are desaturated to palmitoleoyl- and oleoyl-CoA, respectively [31,32]. Feed could affect the Scd1 mRNA expression in different organisms. The high level of SFA palmitic acid (C16:0) detected in abdominal adipose tissue might have stimulated the Scd1 mRNA expression in this experiment. In addition, the lowest expression level of Scd1 detected in birds fed 45% green cabbage would also suggest a low content of SFAs composition. The oleate acid (C18:1) content was reduced in the lipid tissue of SCD1−/− mice [33] suggesting that oleate acid (C18:1) is directly regulated by the expression level of Scd1. Smith et al [34] using piglets demonstrated that subcutaneous linoleic acid (C18:2) could decrease the Scd1 activity by decreasing mRNA expression and (or) catalytic activity. These results explain the positive correlation between the content of palmitic acid (C16:0) and oleate acid (C18:1) with the Scd1 mRNA expression level, and the negative correlation between the content of linoleic acid (C18:2) with Scd1 mRNA expression level.

The high concentration of linoleic acid (C18:2) was always associated with high serum levels of VLDL, TG, FFA, and CM, and lower serum levels of LDL, HDL, and TC, however, it was inversely observed in alpha-linolenic acid (C18:3) when considering its correlation with serum lipid concentrations. It is reported that alpha-linolenic acid (C18:3) and linoleic acid (C18:2) have comparable effects on serum lipid and lipoprotein concentrations, and linoleic acid (C18:2) intake has been suggested to reduce alpha-linolenic acid (C18:3) metabolism [35]. Sarah et al [36] suggested that alpha-linoleic acid intake leads to differential enrichment in LDL, a moderate amount of alpha-linoleic acid was effective in improving lipid profiles of normolipidemic humans. Goyens et al [37] research indicated that low linoleic acid might cause a decrease in medium VLDL, and a high-alpha linolenic acid would cause a decrease in small VLDL.

## CONCLUSION

Green cabbage increased the content of alpha-linolenic acid (C18:3) in adipose tissues. Green cabbage feeding time dependently affected the content of palmitic acid (C16:0), palmitoleic acid (C16:1), linoleic acid (C18:2), and alpha-linolenic acid (C18:3). Considering the content of linoleic acid (C18:2) and α-linolenic acid (C18:3), 30% inclusion of green cabbage might be considered for 30 days fattening of Wanxi White geese.

## Figures and Tables

**Table 1:** Chemical composition and fatty acid content of the feed, including calculated metabolisable energy content

Chemical composition	Percentage of green forage relative to dry matter (%)			

	0	15	30	45
Water content (%)	12.14	12.65	13.78	14.12
Ash (%)	7.11	7.02	6.95	6.82
Crude protein (%)	16.11	15.94	15.72	15.37
Crude fat (%)	1.98	1.92	2.00	1.96
Crude fibre (%)	5.19	5.34	5.69	5.88
Calcium (%)	2.00	1.98	1.96	1.92
Potassium (%)	0.60	0.67	0.72	0.77
Metabolisable energy (MJ/kg)	15.89	15.42	15.21	15.11

**Table 2:** Primer sequences used for reverse transcription-polymerase chain reaction

Gene	Primer sequences 5′-3′	Accession No.	Annealing Temp. (°C)	Product size (bp)
*Scd1*	F: GTTCTCCTCCGCTTCCAG			
	R:CCTTCTCACGGTAGGTCTCA	KF185111.1	53.5	129
*Acsl1*	F: GGGATGTCAGGCAGTATGT			
	R: ATGCTTTCGGTCTTGTCG	XM_005029700.2	54.3	105
*Elovl6*	F: CTGCTCCTTTCATGCTGT			
	R: GTTCGGGTGCTTTGCTTA	NM_001031539.1	57.4	161
*β-actin*	F:CCTCGCTATCGACCTTCCAG			
	R:AGTGAGACGACTCATGCAGC	M26111.1	60.0	139

*Scd1*, stearoly-coA desaturase 1; *Acsl1*, acyl-CoA synthetase long-chain family member 1; *Elovl6*, elongation of long-chain fatty acids family member 6.

**Table 3:** Fatty acids composition in Wanxi White goose was affected by main factors of green forage, feeding time, and adipose tissue (%)

Time	Group	Tissue	C16:0	C16:1	C18:0	C18:1	C18:2	C18:3^1)^
0			23.13^b^	2.42^a^	7.50	47.79^a^	18.64^c^	0.77^c^
10			23.10^b^	2.29^b^	7.69	47.47^ab^	18.87^c^	0.76^c^
20			23.18^b^	2.21^b^	7.53	46.80^b^	19.62^b^	0.85^b^
30			24.11^a^	2.49^a^	7.65	45.26^c^	20.47^a^	0.91^a^
SEM			0.221	0.046	0.159	0.289	0.163	0.021
	0		23.49	2.39	7.54	46.82	19.70	0.79^b^
	15		23.27	2.34	7.52	46.99	19.29	0.78^b^
	30		23.41	2.36	7.73	46.70	19.33	0.83^ab^
	45		23.35	2.33	7.59	46.82	19.27	0.88^a^
SEM			0.208	0.044	0.188	0.339	0.192	0.019
		Abdomi^2)^	23.93^a^	2.26^b^	8.17	46.32^b^	19.18^b^	0.80
		Subcuta^3)^	22.83^b^	2.45^a^	7.08	47.34^a^	19.62^a^	0.84
SEM			0.145	0.038	0.133	0.246	0.135	0.017
p-value								
Time			<0.0001	<0.0001	0.741	<0.0001	<0.0001	<0.0001
Group			0.801	0.828	0.704	0.884	0.140	0.003
Tissue			<0.0001	<0.0001	<0.0001	<0.0001	0.0036	0.097

SEM, standard error of the mean.

1) C18:3: Data listed was the percentage, the p-value was calculated after arcsin transformation. The same to the following tables.

2) Abdomi: abdominal fat tissue.

3) Subcuta: subcutaneous fat tissue.

a–c Values within a column with different superscripts are significantly different, p<0.05.

**Table 4:** Fatty acids composition in Wanxi White goose was affected by cross interactions between green forage, feeding time, and adipose tissue (%)

Time	Group	Tissue	C16:0	C16:1	C18:0	C18:1	C18:2	C18:3
0		Abdomi^1)^	23.11^c^	2.39^bc^	7.37^cd^	47.99^a^	18.64^c^	0.76
		Subcuta^2)^	23.16^c^	2.46^ab^	7.63^bcd^	47.59^a^	18.64^c^	0.77
10		Abdomi	23.18^c^	2.10^d^	8.12^ab^	47.13^ab^	18.89^c^	0.74
		Subcuta	23.03^c^	2.49^ab^	7.26^cd^	47.81^a^	18.85^c^	0.78
20		Abdomi	24.07^b^	2.17^d^	7.86^bc^	46.06^b^	19.16^c^	0.81
		Subcuta	22.29^d^	2.25^cd^	7.20^de^	47.54^a^	20.07^b^	0.89
30		Abdomi	25.38^a^	2.39^bc^	8.53^a^	44.09^c^	20.04^b^	0.91
		Subcuta	22.85^cd^	2.60^a^	6.78^e^	46.42^b^	20.90^a^	0.91
SEM			0.238	0.062	0.217	0.393	0.238	0.030
	0	Abdomi	24.56	2.28	8.10	45.82	19.95	0.83^bc^
	0	Subcuta	22.66	2.47	7.01	47.16	20.16	0.77^c^
	15	Abdomi	24.02	2.22	7.92	46.33	18.90	0.79^c^
	15	Subcuta	22.62	2.41	7.12	47.11	20.12	0.79^c^
	30	Abdomi	24.17	2.18	8.58	45.16	19.27	0.81^bc^
	30	Subcuta	22.85	2.49	7.04	47.50	19.87	0.88^b^
	45	Abdomi	24.09	2.19	8.06	45.73	19.33	0.84^bc^
	45	Subcuta	22.75	2.41	7.17	47.27	19.62	0.98^a^
SEM			0.297	0.077	0.269	0.487	0.275	0.034
p-value								
Time×group			0.730	0.703	0.705	0.964	0.071	0.058
Time×tissue			<0.0001	0.028	<0.0001	0.0015	0.026	0.400
Group×tissue			0.745	0.857	0.520	0.444	0.263	0.043
Time×group×tissue			0.645	0.219	0.500	0.458	0.099	0.118

SEM, standard error of the mean.

1) Abdomi: abdominal fat tissue.

2) Subcuta: subcutaneous fat tissue.

a–d Values within a column with different superscripts are significantly different, p<0.05.

**Table 5:** Gene expression level of Wanxi White goose was affected by main factors of green forage, feeding time, and adipose tissue

Items	Group	Tissue	*Elovl6*	*Acsl1*	*Scd1*
Age					
70			1.909^a^	2.225^a^	3.230^a^
80			1.841^ab^	1.694^b^	1.223^b^
90			1.435^bc^	1.218^c^	0.983^b^
100			1.233^c^	0.866^d^	0.833^b^
SEM			0.147	0.086	0.216
p-value			0.007	<0.0001	<0.0001
	0		1.870	1.687^a^	2.022
	15		1.442	1.519^ab^	1.584
	30		1.560	1.318^bc^	1.372
	45		1.547	1.479^ab^	1.293
SEM			0.172	0.085	0.213
p-value			0.272	0.024	0.072
		Abdomi^1)^	1.558	1.219^b^	0.925^b^
		Subcuta^2)^	1.651	1.782^a^	1.102^a^
SEM			0.562	0.066	0.093
p-value			0.521	<0.0001	0.004

*Elovl6*, elongation of long-chain fatty acids family member 6; *Acsl1*, acyl-CoA synthetase long-chain family member 1; *Scd1*, stearoly-coA desaturase 1; SEM, standard error of the mean.

1) Abdomi: abdominal fat tissue.

2) Subcuta: subcutaneous fat tissue.

a–d Values within a column with different superscripts are significantly different, p<0.05.

**Table 6:** Gene expression level of Wanxi white goose was affected by cross interactions of green forage, feeding time, and adipose tissues

Items	Group	Tissue	*Elovl6*	*Acsl1*	*Scd1*
Age					
70		Abdomi^1)^	1.674^ab^	1.867^bc^	2.268^b^
70		Sbucuta^2)^	2.144^a^	2.583^a^	4.192^a^
80		Abdomi	2.186^a^	1.361^d^	1.114^c^
80		Sbucuta	1.496^b^	2.026^b^	1.332^c^
90		Abdomi	1.480^b^	0.790^e^	0.914^c^
90		Sbucuta	1.390^b^	1.645^cd^	1.053^c^
100		Abdomi	0.892^c^	0.857^e^	0.746^c^
100		Sbucuta	1.574^b^	0.875^e^	0.920^c^
SEM			0.208	0.115	0.312
p-value			0.013	0.003	0.005
Age					
70	0	Abdomi	1.674^bcde^	1.867	2.268
70	0	Sbucuta	2.144^bcd^	2.583	4.192
80	0	Abdomi	3.378^a^	2.122	2.678
80	0	Sbucuta	1.059^de^	1.822	2.501
80	15	Abdomi	1.227^bcde^	1.288	0.874
80	15	Sbucuta	2.424^ab^	2.170	0.868
80	30	Abdomi	2.152^bcd^	1.013	0.478
80	30	Sbucuta	1.134^cde^	2.049	1.470
80	45	Abdomi	1.988^bcd^	1.022	0.427
80	45	Sbucuta	1.369^bcde^	2.064	0.488
90	0	Abdomi	2.257^abc^	0.852	0.950
90	0	Sbucuta	1.202^cde^	2.083	1.194
90	15	Abdomi	1.062^de^	1.156	1.767
90	15	Sbucuta	1.161^cde^	1.609	1.552
90	30	Abdomi	1.453^bcde^	0.518	0.469
90	30	Sbucuta	1.577^bcde^	1.186	0.613
90	45	Abdomi	1.148^cde^	0.636	0.469
90	45	Sbucuta	1.619^bcde^	1.702	0.854
100	0	Abdomi	0.822^e^	0.949	0.960
100	0	Sbucuta	2.421^ab^	1.216	1.430
100	15	Abdomi	0.766^e^	0.731	0.559
100	15	Sbucuta	1.080^de^	0.749	0.588
100	30	Abdomi	1.211^cde^	0.665	0.559
100	30	Sbucuta	1.132^cde^	0.662	0.923
100	45	Abdomi	0.767^e^	1.082	0.906
100	45	Sbucuta	1.664^bcde^	0.872	0.739
SEM			0.415	0.231	0.625
p-value			0.051	0.107	0.998

*Elovl6*, elongation of long-chain fatty acids family member 6; *Acsl1*, acyl-CoA synthetase long-chain family member 1; *Scd1*, stearoly-coA desaturase 1; SEM, standard error of the mean.

1) Abdomi: abdominal fat tissue.

2) Subcuta: subcutaneous fat tissue.

a–e Values within a column with different superscripts are different, p<0.05.

**Table 7:** Serum lipids content in Wanxi White geese fed with different ratio of green forage for different time

Items	Group	VLDL	LDL	TG	HDL	TC	FFA	CM
Age								
70		29.72^a^	1.77	5.11	1.66^a^	4.08	591.90^a^	29.15^a^
80		27.67^b^	1.71	5.13	1.48^b^	4.05	533.64^b^	27.63^b^
90		27.08^b^	1.68	5.38	1.52^ab^	3.90	554.58^b^	27.08^b^
100		26.50^b^	1.69	5.30	1.56^ab^	4.11	543.08^b^	27.45^b^
SEM		0.601	0.026	0.050	0.051	0.101	9.963	0.446
p-value		0.006	0.272	0.499	0.014	0.666	0.0003	0.001
	0	27.66	1.71	5.26	1.57	4.12	563.39	27.61
	15	28.40	1.71	5.24	1.57	4.14	564.68	28.33
	30	28.20	1.72	5.22	1.58	4.08	547.18	28.10
	45	27.40	1.71	5.13	1.53	3.83	555.47	27.68
SEM		0.603	0.026	0.050	0.051	0.101	9.993	0.447
p-value		0.710	0.997	0.935	0.895	0.228	0.670	0.517
Age								
70	0	29.72	1.77	5.11	1.66	4.08	591.90	29.15
80	0	27.04	1.76	5.14	1.49	4.31	540.42	27.32
	15	30.16	1.64	5.19	1.45	4.36	553.70	28.08
	30	27.59	1.76	5.12	1.57	4.00	520.47	28.63
	45	26.13	1.67	5.06	1.39	3.59	522.42	26.53
90	0	25.71	1.64	5.55	1.36	3.74	534.24	25.70
	15	27.99	1.72	5.50	1.58	3.99	572.27	28.95
	30	27.57	1.62	5.29	1.61	4.01	562.90	26.76
	45	27.01	1.73	5.17	1.55	3.86	548.25	26.75
100	0	27.45	1.63	5.35	1.76	4.32	584.35	27.69
	15	25.06	1.72	5.21	1.56	4.10	533.23	26.92
	30	27.46	1.71	5.46	1.44	4.27	504.94	27.15
	45	26.27	1.68	5.22	1.51	3.79	555.51	28.05
SEM		1.201	0.052	0.100	0.101	0.202	19.916	0.891
p-value		0.696	0.884	0.999	0.263	0.703	0.586	0.307

VLDL, very low density lipoprotein; LDL, low density lipoprotein; TG, triglycerides; HDL, high density lipoprotein; TC, total cholesterol; FFA, free fatty acid; CM, chylomicron; SEM, standard error of the mean.

a,b Values within a column with different superscripts are significantly different, p<0.05.

**Table 8:** Correlation coefficient between fatty acid components and related gene expression and serum lipids content

Fatty acid		*Elovl6*	*Acsl1*	*Scd1*	VLDL	LDL	HDL	TG	TC	FFA	CM
C16:0	r	−0.035	−0.013	0.287	−0.054	0.243	0.155	0.026	0.287	−0.167	−0.201
	p	0.627	0.857	<0.0001	0.450	0.0005	0.029	0.719	<0.0001	0.018	0.004
C16:1	r	0.141	−0.057	0.038	0.132	0.112	0.075	−0.059	0.040	0.005	−0.073
	p	0.047	0.421	0.596	0.063	0.114	0.289	0.408	0.575	0.945	0.302
C18:0	r	−0.078	0.057	0.050	−0.144	0.103	−0.095	0.063	0.048	−0.130	−0.130
	p	0.272	0.420	0.481	0.041	0.147	0.183	0.374	0.496	0.067	0.067
C18:1	r	0.076	−0.048	0.265	−0.036	0.007	0.176	−0.178	0.265	−0.039	−0.066
	p	0.288	0.499	0.0001	0.608	0.923	0.013	0.012	0.0001	0.588	0.356
C18:2	r	−0.058	0.043	−0.677	0.200	−0.285	−0.321	0.231	−0.677	0.288	0.369
	p	0.418	0.549	<0.0001	0.005	<0.0001	<0.0001	0.001	<0.0001	<0.0001	<0.0001
C18:3	r	−0.046	−0.039	0.996	−0.304	0.504	0.471	−0.296	0.996	−0.369	−0.482
	p	0.517	0.586	<0.0001	<0.0001	<0.0001	<0.0001	<0.0001	<0.0001	<0.0001	<0.0001

*Elovl6*, elongation of long-chain fatty acids family member 6; *Acsl1*, acyl-CoA synthetase long-chain family member 1; *Scd1*, stearoly-coA desaturase 1; VLDL, very low density lipoprotein; LDL, low density lipoprotein; HDL, high density lipoprotein; TG, triglycerides; TC, total cholesterol; FFA, free fatty acid; CM, chylomicron.
